# Adult Ileocolic Intussusception Following Appendectomy: A Case Report of a Rare Sonographic Finding

**DOI:** 10.7759/cureus.54195

**Published:** 2024-02-14

**Authors:** Abdulaziz A Hussein, Awadia G Suliman

**Affiliations:** 1 College of Medicine, University of Medical Science and Technology, Khartoum, SDN; 2 Faculty of Radiological Sciences and Medical Imaging, Alzaiem Alazhari University, Khartoum, SDN; 3 Department of Diagnostic Radiology Technology, College of Applied Medical Sciences, Taibah University, Al-Madinah Al-Munawarah, SAU

**Keywords:** case report, ultrasound, appendectomy, ileocolic intussusception, adult intussusception

## Abstract

Adult intussusception is a rare condition characterized by the telescoping of one segment of the intestine into an adjacent segment. Prompt recognition and intervention are crucial due to the potential for serious complications. The present case is of adult ileocolic intussusception in a 47-year-old male patient who underwent appendectomy three weeks prior. The patient presented with constipation, lower abdominal pain, and vomiting. A transabdominal ultrasound revealed characteristic sonographic features, including a target appearance at the transverse view and the pseudokidney sign of the longitudinal view associated with the presence of reactive lymph nodes. Doppler ultrasound indicated no internal flow, suggesting possible ischemia. This case highlights the role of ultrasound in the initial evaluation of adult intussusception and emphasizes the need for further imaging modalities for detailed anatomical evaluation and lesion identification.

## Introduction

Adult intussusception, a condition characterized by the telescoping of one segment of the intestine into an adjacent segment, is an uncommon entity that poses significant diagnostic challenges. Unlike its pediatric counterpart, adult intussusception is a relatively rare occurrence, accounting for only 5% of all intussusception cases, with an estimated incidence of 1-4 per 1,000,000 population per year [1,2]. Despite its rarity, adult intussusception demands prompt recognition and intervention due to the potential for serious complications, including bowel ischemia and perforation [3,4]. Unlike pediatric cases, this intussusception type is frequently associated with an identifiable pathology, such as benign or malignant neoplasms, postoperative adhesions, Meckel's diverticulum, polyps, or inflammatory bowel disease [5,6]. An erect abdominal radiograph is considered the initial imaging technique for identifying intestinal obstruction, perforation, or ascites. A transabdominal ultrasound is an imaging modality used to evaluate adult intussusception. It can provide real-time dynamic imaging and aid in the identification of the telescoping bowel segments. Here, the report described a case of adult ileocolic intussusception of a patient with a close history of appendectomy. 

## Case presentation

**Figure 1:**
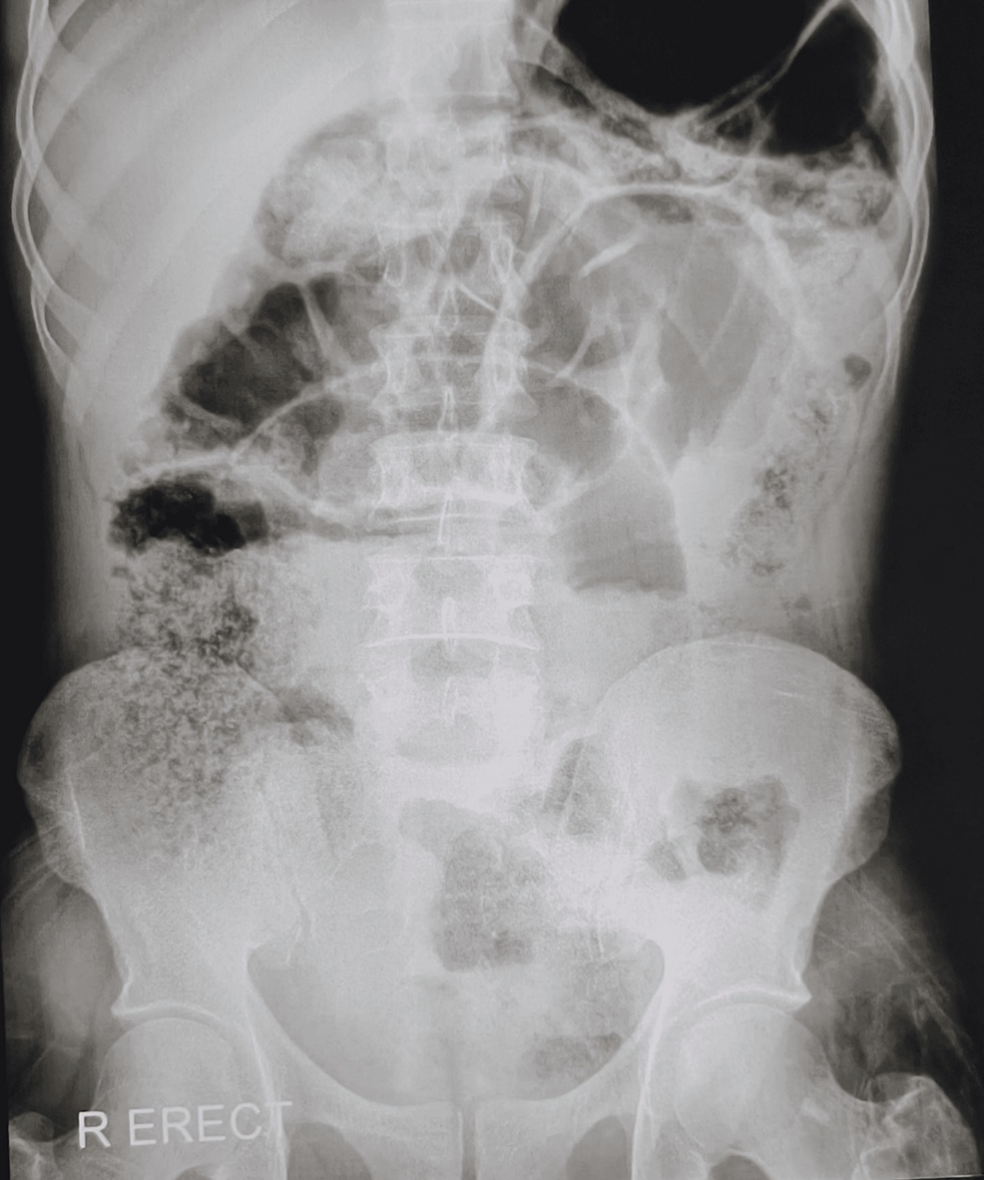
Radiographic image of the abdomen (AP - erect) shows excessive bowel gases and impacted fecal material, along with dilated distal small intestines, suggesting intestinal obstruction.

A 47-year-old male patient was referred from the ER following a surgical appendectomy three weeks ago for histologically confirmed suppurative appendicitis without perforation. The patient reported experiencing renewed right iliac fossa pain and constipation for one week, along with worsening lower abdominal pain and vomiting. A physical examination revealed a palpable sausage-like mass in the right iliac fossa, which was tender and guarded upon probing. No abnormalities were observed during the rest of the physical abdominal pelvic examinations. The laboratory results showed a hemoglobin level of 12.6 g/dl, elevated total leukocyte counts (14,630/µL) with a particularly high percentage of neutrophils (77.3%) (Table 1). An abdominal X-ray displayed excessive bowel gases and impacted fecal material, along with dilated small intestines, suggesting intestinal obstruction without the presence of an obvious soft-tissue mass lesion (Figure 1).

**Table 1:** Laboratory investigations

Parameters	Results	Reference values
Hemoglobin	12.6 g/dl	13 -17 g/dl
Platelets	512	250 – 400*109/L
WBCs	14,630/µL	4000-10,500/µL
Neutrophils (relative percent)	77.3	40 – 70 %
Lymphocytes (relative percent)	21.2	20 – 40 %
Monocytes (relative percent)	1.4	2 – 8 %
ESR (erythrocyte sedimentation rate)	55 mm/hr	Male < 50 years: < 15 mm/hr
ALT (alanine transaminase)	39	10 - 49 U/L
Creatinine	0.9 mg/dl	0.6 - 1.2 mg/dl
Potassium (K+)	4.86 mg/dl	3.5 - 5.3 mg/dl
Lipase	25 U/L	12-53 U/L

**Figure 2:**
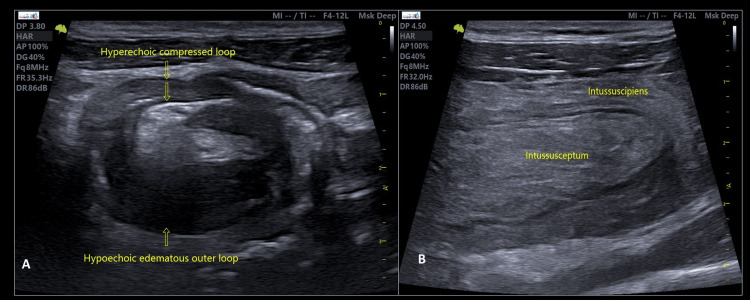
A) Transverse sonographic image of intestinal intussusception, the inner fat-containing mesentery entrapped within compressed hyperechoic bowel loops. This classic sign resembles the pseudokidney sign. B) Longitudinal section showing the navigation of intussuscipiens represents the received bowel and the involved central area of alternating echogenicity. Intussusceptum represents the compressed prolapsed bowel segment (gut within the gut).

**Figure 3:**
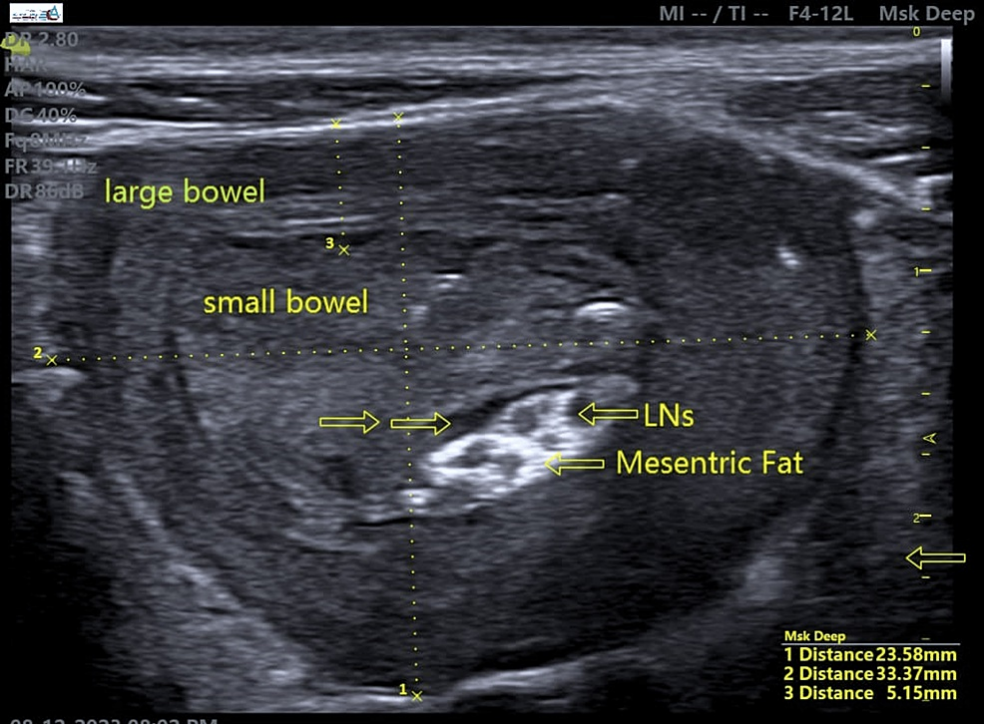
A transverse view of ileocolic intussusception characterized by a rounded, concentric alternating structure in the right lower abdominal side, with the hyperechoic eccentric area representing mesenteric fat, occupied lymph nodes, and adjacent reactive entrapped fluid.

**Figure 4:**
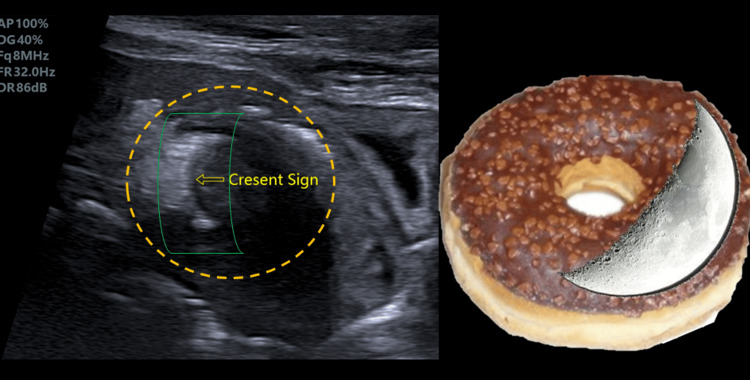
The crescent-in-doughnut sign represents the echogenicity area for inflamed mesentery fat enclosed within the intestinal intussusception compartment.

**Figure 5:**
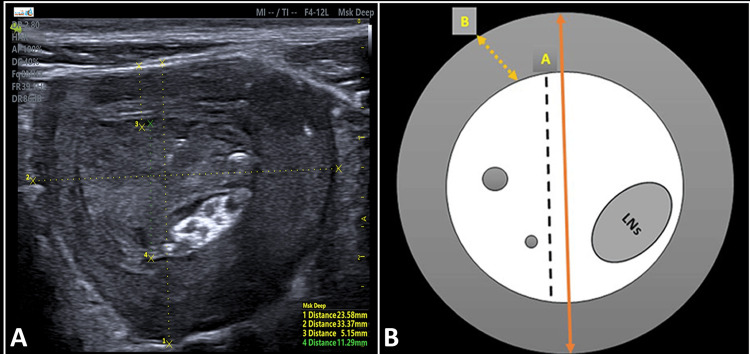
A) The ultrasound measurements that support and distinguish the diagnosis of Ileocolic intussusception from other small bowel types including the inner central core diameter (11.29 mm) and the outer wall thickness (5.15 mm). B) Schematic demonstration of the core-to-wall index (A/B) ratio of the inner core diameter to the outer wall thickness (ratio: 2.20 > 1). Additionally, the presence of reactive lymph nodes (LNs) within the large AP diameter (23.58 mm) and transverse diameter (33.37 mm) also contribute to an accurate diagnosis.

**Figure 6:**
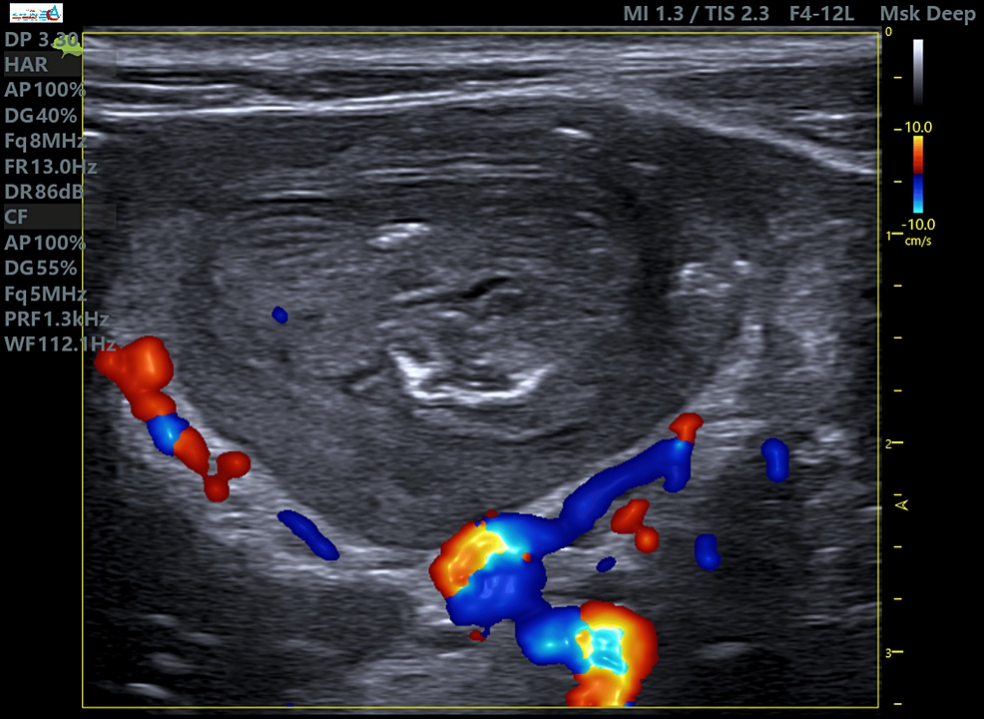
The absence of the internal vascular flow on applied color Doppler within the intussusception compartments (even with applying high-flow sensitive Doppler measurements and luminance Doppler setting). Moreover, trapped fluid within is a concerning sign, which may indicate ischemia or necrosis and potentially increase the risk of enema reduction failure.

**Figure 7:**
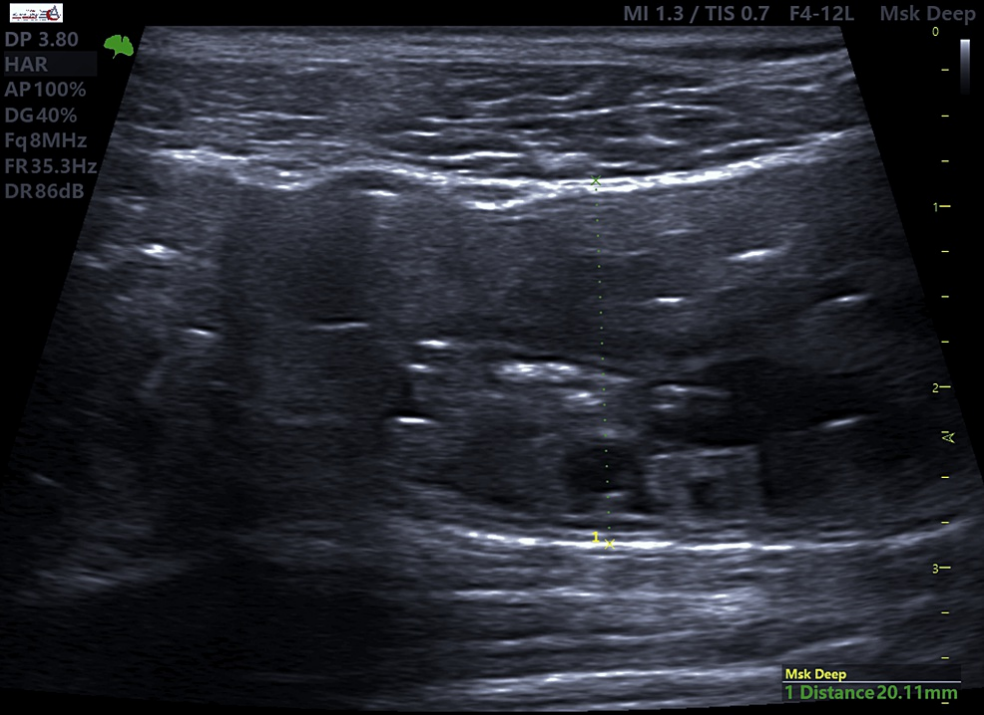
Sonographic image of proximal dilated and hypo-peristaltic bowel loops (20.11 mm).

Subsequent ultrasound scans of the abdomen and pelvis revealed the presence of a rounded structural mass on the right side of the abdomen, exhibiting a pseudo-kidney/doughnut/target appearance and possible peristaltic gut layer signature (Figures 2, 3, 4) (Video 1). The ultrasound measurements were consistent with ileocolic intussusception. The large measurements, presence of entrapped reactive lymph nodes (LNs), and free fluid supported this diagnosis (Figure 5). Color Doppler imaging did not detect internal flow within the intussusception compartments, indicating possible ischemia and reducing the likelihood of a successful enema study (Figure 6). Proximal dilated and edematous bowel loops were visualized (Figure 7). No stump or inverted retained appendix was observed, suggesting post-operative adhesions as the most likely etiological cause, which was confirmed by the surgeon. The rest of the organs in the abdomen were normal, except for a right renal cortical cyst and prostatic calcifications.

**Video 1:** Real-time B-mode sonographic scan for Iliocolic intussusception.

## Discussion

A transabdominal ultrasound is the primary imaging modality used to evaluate adult intussusception. It can provide real-time dynamic imaging and aid in the identification of the telescoping bowel segments. Typical sonographic findings include a target or doughnut sign, characterized by concentric hypoechoic (outer - intussuscipiens) and hyperechoic rings (inner - intussusceptum). The central hyperechoic area corresponds to the mesenteric fat and vessels within the intussusception [7,8]. In addition to the target sign, other sonographic features that may be observed include a pseudokidney sign (resembling a kidney-shaped structure), a sausage-shaped mass, or a whirlpool sign (indicating twisting of the mesentery) [9,10]. Color Doppler ultrasound can provide information on the vascularity within the intussusception and help assess the prediction of ischemia or infarction [8]. While ultrasound is valuable as an initial diagnostic tool, it has some limitations. The accuracy of ultrasound in detecting the cause of intussusception, such as identifying specific underlying lesions, is relatively low compared to other imaging modalities like CT or MRI. Furthermore, ultrasound may be limited by factors such as bowel gas or obesity, which can hinder visualization and interpretation of the images [11]. In cases where the diagnosis remains inconclusive or further characterization is required, additional imaging modalities such as CT or MRI are often employed. CT scan, in particular, is considered the gold standard imaging modality for adult intussusception due to its ability to provide detailed anatomical information, assess for complications, and identify the cause of intussusception [12]. However, it is also important to consider the associated radiation exposure when considering the diagnostic benefits of CT.

## Conclusions

In summary, ultrasound plays a crucial role in the initial evaluation of adult intussusception, offering real-time dynamic imaging and aiding in the detection of characteristic sonographic features of ileocolic intussusception as a post-appendectomy complication. It is advised to use Doppler integration to assess intestinal blood perfusion and rule out ischemia or necrotic changes that might be causing perfusion. However, this utility may be limited in cases requiring a detailed anatomical evaluation or the identification of specific underlying lesions, for which CT or MRI may be necessary.
